# Consent in pregnancy - an observational study of ante-natal care in the context of Montgomery: all about risk?

**DOI:** 10.1186/s12884-021-03574-2

**Published:** 2021-02-01

**Authors:** Jacqueline A. Nicholls, Anna L. David, Joseph Iskaros, Dimitrios Siassakos, Anne Lanceley

**Affiliations:** 1 EGA Institute for Women’s Health, Faculty of Population Health Sciences, University College London, Medical School Building, 74 Huntley Street, London, WC1E 6AU UK; 2 Elizabeth Garrett Anderson Wing, University College London Hospital NHS Foundation Trust, 25 Grafton Way, London, WC1E 6DB UK; 3 NIHR University College London Hospitals Biomedical Research Centre, Research & Development, 149 Tottenham Court Road, London, W1T 7DN UK; 4 Wellcome / EPSRC Centre for Interventional and Surgical Sciences, University College London, Gower St, Bloomsbury, London, WC1E 6BT UK

**Keywords:** Consent, Choice, Consultations, Values, Autonomy, Ante-natal care, Montgomery, Clinical risk, Invasive procedures, Cesarean section

## Abstract

**Background:**

How to best support pregnant women in making truly autonomous decisions which accord with current consent law is poorly understood and problematic for them and their healthcare professionals. This observational study examined a range of ante-natal consultations where consent for an intervention took place to determine key themes during the encounter.

**Methods:**

Qualitative research in a large urban teaching hospital in London. Sixteen consultations between pregnant women and their healthcare professionals (nine obstetricians and three midwives) where ante-natal interventions were discussed and consent was documented were directly observed. Data were collectively analysed to identify key themes characterising the consent process.

**Results:**

Four themes were identified: 1) Clinical framing - by framing the consultation in terms of the clinical decision to be made HCPs miss the opportunity to assess what really matters to a pregnant woman. For many women the opportunity to feel that their previous experiences had been ‘heard’ was an important but sometimes neglected prelude to the ensuing consultation; 2) Clinical risk dominated narrative - all consultations were dominated by information related to risk; discussion of reasonable alternatives was not always observed and women’s understanding of information was seldom verified making compliance with current law questionable; 3) Parallel narrative - woman-centred experience – for pregnant women social factors such as the place of birth and partner influences were as or more important than considerations of clinical risk yet were often missed by HCPs; 4) Cross cutting narrative - genuine dialogue - we observed variably effective interaction between the clinical (2) and patient (3) narratives influenced by trust and empathy and explicit empowering language by HCPs.

**Conclusion:**

We found that ante-natal consultations that include consent for interventions are dominated by clinical framing and risk, and explore the woman-centred narrative less well. Current UK law requires consent consultations to include explicit effort to gauge a woman’s preferences and values, yet consultations seem to fail to achieve such understanding. At the very least, consultations may be improved by the addition of opening questions along the lines of ‘what matters to you most?’

## Background

Pregnant women’s rights to share in decisions concerning their care are central to woman-centred maternity care. In the UK these rights are affirmed by professional guidance [1] and, since 2015, by the UK Supreme Court following the landmark decision of Montgomery v Lanarkshire Health Board [2] clarifying the nature of the relationship between healthcare professional (HCP) and patient on information disclosure.

Nadine Montgomery’s claim for loss, injury and damages sustained by her baby son who developed cerebral palsy were based on the ground that no ordinarily competent obstetrician acting with reasonable skill and care would have: allowed a diabetic woman of short stature with macrosomic foetus in “early trial of labour” whose foetal heartbeat was grossly abnormal to continue in labour and attempt a vaginal delivery; or fail to consider offering delivery by caesarean section. Echoing a decision taken by the Australian Courts in the 1990’s [3] the decision in *Montgomery* and subsequent cases [4–8] reflects increasing insistence that patient autonomy is fully implemented via a process of real dialogue between HCPs and their patients. By requiring a HCP to advise patients about alternatives and risks which may be *material* to their *particular* individual circumstances it endorsed a patient’s central role in decision-making. This fully dialogical, tailored approach to information giving in which both parties participated in discussion is inherently challenging in practice not least because what makes a risk or intervention acceptable to one patient may render it unconscionable to another and the broader factors which affect patient’s decision-making are highly individual.

Nevertheless as a matter of law a doctor,

*‘has a duty to take reasonable care to ensure that the patient is aware of any material risks involved in any recommended treatment and of any reasonable alternatives or variant treatments’* [2] (para 87)

The test of materiality is ‘whether, in the circumstances of the particular case, a reasonable person in the patient’s position would be likely to attach significance to the risk, or the doctor is or should reasonably be aware that the particular patient would be likely to attach significance to it [2] (para 87). Whether a risk is regarded as material depends on the patient’s perspective rather than the HCPs.

Merely providing patients with information is insufficient yet limited evidence indicates this is often what happens in practice [9], possibly even more so since the Montgomery ruling. Consultation dialogues should ensure that patients understand the options available and are supported in making meaningful choices by being provided with information on alternatives and risks relevant to *them*. However, assessing what might be material to a particular patient requires careful and skilful exploration of their personal expectations and values if autonomous fully shared decision-making is to be supported.

Although limited evidence [9, 10] indicates that some HCPs have changed their practice following the Montgomery ruling whereas others have not, there is a paucity of evidence concerning how HCPs apply current legal guidance in clinical consultations.

This observational study aimed to examine how consent unfolds and decisions are made in the social interaction of ante-natal consultations for interventions.

## Methods

### Design

This exploratory qualitative study combined the ethnographic methods of participant observation and semi-structured interviews with immersion into the local world of the consent encounter. A distinction of the ethnographic paradigm is its potential to produce naturalistic, transferable generalisations rather than statistical types of generalisability [11]. Only the observational data are reported here, the interview data having been reported previously [9]. We report in accordance with international accepted guidelines (COREQ) [ 12] and in a manner that calls upon readers to actively engage in assessing its value beyond its contextual confines.

### Study participants

Participants were recruited from an urban teaching hospital providing ante-natal healthcare to approximately 6500 women annually. Eligible consultations involved pregnant women who met all the following inclusion criteria: Aged > 18 years; First or subsequent pregnancies; Able to understand spoken and written English; Managed in consultant or midwife-led ante-natal clinic. HCPs were practicing obstetric doctors or midwives responsible for managing consultations for healthcare interventions such as Cesarean section, cervical cerclage, and prenatal diagnosis procedures. All observed consultations involved the participants giving fully informed written consent.

A purposive convenience sample of eligible women were identified by the HCP team from clinic lists aimed to ensure maximum variation of consultations with regards to setting; intervention being discussed; and member of staff taking consent. In this snapshot observation study of consent, eligibility and study entry were independent of knowing if a woman had or had not had previous antenatal discussions about consent to care interventions. Participants provided informed written consent to the research in advance of the clinical discussion.

### Data collection

Participant observation is used to understand the world as it is seen and experienced by those acting within it [13]. The aim was to observe the clinical consent narrative of each clinical encounter, to understand how care decisions are communicated, and to gain an overall feel and sense of woman-HCP understanding and interaction in the context of consent. Consultations were observed by the researcher (JN) who has a background in medical law and social science research. The researcher sat in a suitably discreet position in the consultation room, so as not to disturb the natural flow of the clinical encounter. Field notes were taken in accordance with guidelines provided by Spradley [14]. As per the ethnographic method the researcher made written notes in free style prose during the consultation noting information such as non-verbal communication, distress, engagement, tensions, and the balance of communication and rapport between the parties.

Family members were present in some consultations. Socio-demographic details were collected directly from participants and medical information from their medical records.

### Data analyses

Field notes were transcribed and anonymised. The Framework Method [15] was used to analyse the notes to allow identification of commonalities and differences in the data before considering relations between different parts of the data.

All transcribed notes were thoroughly read several times. Following data familiarisation the researcher read each transcript line by line and allocated a label (‘code’) to describe what the researcher interpreted as important. Two researchers independently coded the first two transcripts and a final set of codes was agreed to apply to all of the transcripts. Codes were grouped together into categories to form a working analytic framework. A framework matrix of each category was created and the data from each transcript were charted into the matrix. Themes were generated from the data by making connections within and between participants and categories. The transcripts were reread and all the data re-examined for fit with the coding matrix. Themes were identified which captured the overall meaning and substance of the data. Reliability of the coding was established through independent coding of 20% of the transcripts and differences discussed until agreement was reached.

The researchers were all HCPs trained in the international Good Clinical Practice (GCP) ethical, scientific and practical standard to which all clinical research is conducted.

## Results

Sixteen consultations were observed lasting 25–75 min. To protect participants confidentiality we report aggregate demographic data (age range = 25–51 years; median 36.5 years; gestational age range = 12–39 weeks, media*n* = 28.5 weeks; parity - nulliparous n = 2, primparous *n* = 13, multiparous n = 2). Table 1 reports the setting and nature of encounters.

**Table 1:** Setting and nature of Observed consultations

Participant	Setting CL = consultant led clinic MW = midwife led clinic	Nature & context of Consent decision
1	CL Ante-natal clinic	Category 4 Elective Cesarean section for history of precipitate vaginal delivery
2	CL Fetal medicine clinic	Cervical Cerclage due to previous late miscarriage
3	ML Ante-natal clinic	Category 4 Elective Cesarean Section for breech presentation
4	ML Ante-natal clinic	Category 4 Elective Cesarean Section for breech presentation
5	ML Ante-natal clinic	Category 4 Elective Cesarean Section for previous symptomatic 3rd degree tear
6	CL Ante-natal clinic	Category 4 Elective Cesarean Section for previous symptomatic 3rd degree tear
7	CL Ante-natal clinic	Category 4 Elective Cesarean Section for previous symptomatic 3rd degree tear
8	Labour Ward	Category 4 Elective Cesarean Section, maternal request, IVF pregnancy
9	CL Ante-natal clinic	Category 4 Elective Cesarean Section for breech presentation
10	CL Fetal Medicine Unit – midwife consent + team	IVF pregnancy. Amniotic drain for fetal growth restriction
11	CL Fetal Medicine Unit – midwife consent + team	Prenatal diagnosis: Chorionic villus sampling for cystic fibrosis
12	CL Fetal Medicine Unit – midwife consent + team	Ultrasound guided Feticide (intracardiac potassium chloride) for fetal abnormality
13	CL Ante-natal clinic	Category 4 Elective Cesarean Section
14	Labour Ward	Category 2 Cesarean Section for failure to progress, pathological CTG
15	Labour Ward	Category 3 Elective Cesarean Section for early onset fetal growth restriction
16	Labour Ward	External Cephalic Version

Four themes were identified which illustrate how consent decisions are played out in context and subject to a number of interplaying influences at any one time. Themes are not entirely distinct and can overlap and/or interrelate with each other as illustrated in the excerpts from the field notes presented in Tables 2, 3, 4 and 5.

### How the consultation was framed – priority of clinical decision narrative

Most consultations began with a HCP welcoming the woman followed by specific questions about a woman’s health and confirmation of the purpose of the consultation. Almost all consultations framed the purpose of the consultation in clinical terms with predominance placed on medical skills and knowledge and that tended to delineate subsequent discussion. For example, it was common for a HCP to ask ‘*have you thought about what you want to do’* and then proceed to explain the clinical merits and demerits of different options or to say *‘I need to listen to the baby’s heartbeat’* or *‘we need to decide whether you want to try for a vaginal delivery or plan for another section’.*

Women mostly responded to this clinical framing by providing further ‘clinical’ description of what had happened since their last consultation - *‘my blood pressure was high’ ‘I’m getting a lot of leg cramps’.* What was striking about these discussions was that the precedence given to clinical considerations at the beginning of the consultation set the limits and tone for the way in which the consultation developed. This was reinforced by the construction of a woman’s situation in terms of describing clinical risk. (Table 2). No consultation included an explicit effort at the outset to assess a woman’s values and preferences by, for example, exploring with a woman what was important to her.

**Table 2:** How the consultation was framed – priority of clinical decision narrative

	***Field note from pre-term birth clinic.*** We learn from the HCP that A has had recurrent miscarriages and the issue is whether she wants to undergo cervical cerclage to minimise her risk. The possibility of cerclage has previously been raised with A.
**HCP**	‘*what we need to decide today in whether you want to have a little operation to see if we can prevent another miscarriage or whether you want to hope for the best’*. A friendly discussion ensues in which HCP summarises A’s situation including her past history of miscarriages and checks *‘have I missed anything’*
**A**	smiling says *‘no I just don’t know what’s the best thing to do’* but doesn’t want another miscarriage.
**HCP**	asks if she’s talked it over with husband
**A**	*‘yes endlessly’*
**HCP**	explains option of doing nothing and putting in stitch – explains how stitch can reduce risk of pre-term birth – but no guarantees; very down to earth – factual but kind Explains 2 types of stitch differences – McDonald and Shirodkar –
**A**	very attentive – moves to edge of chair…seems to be following but very tense
**HCP**	lots of eye contact seems to realise A nervous - explains done under general anaesthetic
**A**	*‘I’m terrified of surgery*’
**HCP**	Explains procedure, draws diagram – legs in supports - ‘off to sleep, hold cervix in place with 2 little holder ties like purse strings
**A**	grimaces, nods
**HCP**	lot to take on board - any questions no *– ‘Have a little think about it whilst I go and get a form’*
**A (to researcher)**	*‘I don’t know what to do………..I’m not medical at all so it’s not like I understand what’s best….but she’s so nice she gives me confidence’*

As the excerpts in Table 2 indicate, many women sought an opportunity to voice their often traumatic, previous childbirth experience as a prelude to the decisions they needed to make, but sometimes appeared frustrated as HCPs sought to focus on the current pregnancy.

### Pre-eminence of clinical risk

Discussion of clinical risk dominated all consultations. We found considerable variability in the approaches taken to discussing risk and alternative care options (Table 3). Most consultations included the provision of large amounts of ‘standard’ information and extensive disclosure of risks but whereas some HCPs disclosed all serious and frequent risks, others were more selective. Occasionally some risks were described as ‘*never a problem’.* HCPs also varied in relation to whether they discussed the risks of not intervening actively, for example the risk of a normal vaginal birth in the case of a recommended Cesarean section.

**Table 3:** Pre-eminence of Clinical Risk

	**Excerpt 1:** ***Field note from Labour Ward. Consent for External Cephalic Version*** This is C’s second pregnancy. She found out through a telephone call from clinic midwife a couple of days earlier (Friday afternoon) but the call was brief and *they ‘just said they could try to turn baby to make birth easier’*. Since then she has been *‘stressed all weekend’ and ‘Googling madly’* and is very worried.
**C**	sitting on bed is quite apprehensive and says she doesn’t really know the score – Dr. pops in says someone will be back. Waiting for 20 min or so – people popping in – seems busy and C very tense, fidgety
**HCP**	enters room with junior Dr. + medical student + midwife – asks C if she understands what they want to try to do – friendly but hurried – all 3 standing looking at C on bed
**C**	*‘sort of’ ‘not had very much information’* – explains phone call – nothing else
**HCP**	standing - explains purpose of ECV and how procedure done – pressure for 15 mins – might hurt
**C**	listens intently - looks terrified – HCP appears not to notice
**HCP**	*have risks been explained?*
**C**	*‘no, looked at Google’* very confused
**HCP**	doesn’t seem to realise how ill-prepared C is. Explains that there are very few risks – mainly that it won’t work no other risks mentioned (? Why not) (should there be discussion of Cesarean Section?) Asks C if she has any questions
**C**	wants to know how likely it’ll work – not really answered. Very rushed
**HCP**	asks C to sign consent form – form signed leaves says they’ll be back as soon as they can
**C to researcher**	says she hopes she’s doing the right thing – I get the sense she feels a bit trapped by coming to the Labour Ward as though she can’t change her mind
**Impression**	C needs more chance to discuss – this does not really constitute proper consent in any way – it’s been very fast (Labour ward is busy) and there’s been no discussion of pros and cons
	**Excerpt 2:** ***Field note from Labour Ward/Ante-natal clinic. Consent for Cesarean Section*** T is a primiparous womanwith an IVF pregnancy. She is being seen in ante-natal clinic but admitted to Labour ward for monitoring (due to raised glucose readings) and potentially a CS in a few days
**HCP**	explains CS procedure – details of practicalities of anaesthesia – T seems very relaxed Explains risks of CS very thoroughly & clearly – infection/antibiotics; deep vein thrombosis/TED Stockings; bleeding; bladder injury; damage to other structures/repair – very reassuring – checks T seems comfortable physically sitting. HCP goes slowly and asks T if any questions several times *– is that clear? does that make sense?* - seems to be trying to make sure T is really engaged without alarming her or making her feel pressured into asking questions
**T**	looks attentive but not sure if she’s really taking it in – seems to be saying yes to everything! Lots of laughter very excited by birth – seems very relaxed about risks

Risks were usually communicated using ratio numbers or percentages which some women struggled to understand in relation to *their* pregnancy. All HCPs invited women to ask questions and some women asked how likely this risk is to happen to *me*. HCPs usually responded by rephrasing or repeating the numerical information. In one consultation a HCP asked the woman how she felt about the risk but most consultations omitted any exploration of a woman’s attitude to risk, either in general or in relation to a specific risk. There was no exploration of the value that women, and their family when present, attached to specific advantages and disadvantages of interventions.

All of the women had previously had some discussion with a HCP about the decision for intervention. Most were given time during the consultation to think about their preferred choice and were invited to ask questions but women’s understanding was not formally assessed. We observed some women who appeared unwilling or unable to engage despite the HCP’s encouragement to do so. For a small number of women the consent process was a more cursory matter in which a woman’s engagement was minimally facilitated (Table 3, excerpt 1).

Discussion of alternative options was variable and in some cases no alternative course of action was discussed. For example, a woman who had had a previous third degree tear was told *‘of course you’ll have to have a CS’* without further discussion. The field note of this consultation records the woman as *‘looking baffled’* in response to mention of risks to the bladder and when interviewed subsequently the woman confirmed she ‘*hadn’t a clue what all that was about’*.

### Woman-centred narrative

A key finding was that in parallel with the clinical and risk dominated narratives many women had other concerns based on their identity as a woman first and as a pregnant woman second which significantly influenced their decision-making - a women-centred narrative.

Some women referred extensively to a traumatic previous experience. They sought acknowledgment of this trauma and a recognition that it was a significant influence on the current pregnancy and something they were anxious to avoid repeating – ‘o*bviously I want to do what’s best for the baby but I don’t want to go through that again’.* HCPs varied in their response. Some HCPs barely acknowledged the previous experience preferring to focus on the current pregnancy to the evident frustration of the woman concerned and this apparent ignoring of their experience shaped the remainder of the consultation (Table 5).

Family influences on decision-making were also observed. For example we observed a consultation involving a woman with an apparently supportive partner for whom a second CS was their preferred option, until the HCP indicated that the increased risk associated with repeat CS was a factor to be considered if they planned a large family. An evident coolness descended on the consultation and in the ensuing discussion the partner discouraged the woman verbally and non-verbally from consenting to a CS but this went undetected by the HCP.

We observed several consultations in which a woman had made a decision on clinical risk grounds but later in the consultation became aware of other factors which changed that decision. For example, a woman who had decided that an elective CS was her preferred option was visibly distressed when told that the elective operation might need to take place at another hospital for workload reasons. (Table 4, excerpt 2) The HCP mistakenly thought she was concerned about the risks of having a CS. She subsequently confirmed to the researcher that she was planning to forego an elective CS and *‘take my chance’*.

**Table 4:** Woman-centred narrative

	**Excerpt 1:** ***Field note from ante-natal clinic for breech presentation*** This is S’s second pregnancy. She is accompanied by her husband. Discussion of elective Cesarean section or trial of labour.
**HCP**	welcomes and explains need to decide whether S wants to opt for a Cesarean section or *‘risk’* a trial of labour. *‘you need to think about the risks and benefits of both decisions and decide what’s best for you’*
**S**	says she thinks a Cesarean would be less risky and wants to feel she has a plan this time
**HCP**	*‘well having a CS would be the safest route’*
**S**	nods – seems to be happier
**HCP**	Asks if S has thought about whether she wants more children
**S**	looks uncomfortable – *‘possibly’*
**HCP**	explains that once she’s had two CS then next birth would need to be CS but she needs to be aware that the risks increase with each subsequent pregnancy Extensive discussion of risks
**Husband**	sits forward on to edge of chair seems a bit upset – says natural birth is *‘usually better’* and thinks *‘we’* would be better with that; quite dominant and almost aggressive – avoids eye contact
**S**	avoids eye contact and doesn’t say anything – marked ‘tension’ in room; very subdued looks upset
**HCP**	says she can sign the consent form now but can change her mind at any point
**S**	says she’ll do it now; husband looks very cross
**HCP**	asks S and husband if either have any more questions
**S**	No further questions; thanks HCP. Husband is silent – doesn’t say goodbye
**Impression**	HCP – very kind, factual – initially good rapport between the three of them but very different at end. HCP doesn’t seem aware of this. Subsequently, despite having signed the consent form, the woman confided to the researcher that she was very stressed didn’t know what to do *‘I think I should have it (CS) but if I do he’ll say I’m making a decision on the size of our family’*
	**Excerpt 2:** ***Field note from ante-natal clinic for breech presentation*** Relaxed friendly consultation with extensive discussion of risks – L seemed very contented and elective Cesarean Section agreed on. Towards the end of the consultation the following discussion occurred:
**HCP**	one practical thing to think about is that we can’t tell you where you’ll have it. explained to L that her consent was for the CS but not the site – might not be at XXX as about 6 people a week sent to YYY hospital because of bed space
**L**	looked a bit shocked by this – didn’t say very much but seems deflated – get the impression this might be a game-changer for her? HCP doesn’t seem to have logged L’s reaction
**L**	thanks HCP – takes consent form (which she had signed earlier in the consultation) and outwardly still positive but looks dejected
**L to researcher**	Subsequently confirms to researcher that she will not have an elective Cesarean section but would rather ‘take her chance’ at XXX hospital and ‘would never’ go to YYY

### Cross-cutting narrative – genuine dialogue

The previous narratives were strongly influenced by the extent to which the interaction between the clinically dominated narrative initiated by HCPs and the personal experience narrative offered by pregnant women was facilitated by genuine dialogue and sharing of decision-making. We observed considerable variability in the nature of shared decision-making in terms of the roles played by HCPs and women. Some consultations were characterised by a strong dialogical exchange in which both parties appeared to exchange ideas freely. Conversely, in other consultations the role of the HCP was essentially that of an information provider. In some cases women tried to be more actively involved but were discouraged from doing so. (Table 5).

**Table 5:** Cross-cutting narrative – genuine dialogue

	***Field note from ante-natal clinic for elective Cesarean Section*** N is a woman in her second pregnancy having suffered a traumatic first delivery which included a third-degree tear (obstetric anal sphincter injury). She is not sure whether to try for another vaginal delivery or have an elective Cesarean section. She has her first child in pushchair with her.
**HCP**	tells her to come in sit down – standing shuffling papers – little eye contact.
**N**	seems distrustful. Sitting forward supporting chin with hand. Starts to tell HCP about her first delivery - lots of symptoms – major problem with tear - bowel interference bleeding from bowel – magnesium tablets *–* ‘it’s been really, really awful’ - seems to want to tell him about her previous experience (he doesn’t seem aware) He doesn’t seem interested but this clinic is packed – heaving waiting room
**HCP**	a bit distracted – struggling with computer … little eye contact. Limited rapport *you didn’t want a sweep* – sounds a bit accusatory
**N**	*‘no’*
**HCP**	*‘this is a consent form – basically we tell you the possible rare risks ….and then you sign this for us’.*oLooking at form goes through risks - any op has risks, bleeding/transfusion, infection/antibiotics, bladder ‘very rare’ ‘no big deal’, blood clots TED stockings, no other procedures planned but repair any damage – if can’t at time then later. Very, very fast
**N**	doesn’t say anything or acknowledge – defensive arms folded – looks unhappy distracted by fractious toddler gives him a drink (researcher tries to distract toddler – N smiles at researcher) Knock on door – colleague enters – asks advice
**HCP**	leaves room says he’ll be back
**N to researcher**	says he’s not listening to what she’s saying – gives toddler drink – researcher encourages her to ask HCP questions
**HCP**	returns – *where were we? ‘Oh yes’.* explains about bikini line incision HCP – passes N form says this is my signature you sign here
**N**	hesitates, looks at form appears to be reading – seems very unhappy? sullen
**HCP**	seems oblivious – busy with computer screen Long pause HCP – tries to take form from N
**N**	hangs on to form – ‘tussle over table’ – she hasn’t signed it – she doesn’t let go of the form
**HCP**	*’anything you don’t understand’*
**N**	queries section on other procedures which might be needed
**HCP**	no extras planned so doesn’t apply
**N**	reads form further – several minutes pass
**HCP**	asks if anything else – impatient
**N**	asks him what he advises – have Cesarean section or try natural birth says *‘I’m not sure’*
**HCP**	*‘I thought you were sure’* – kindly tone but seems impatient? confused by her apparent wavering (? not sure why they haven’t discussed alternative in detail?)** Explains first delivery big achievement – second should be easier – induction + smaller baby. Risk from every direction – given that she had lots of problems after delivery CS is the best option for her.
**N**	looks unconvinced - HCP doesn’t notice
**HCP**	books date – looks at form and says *‘good you’ve signed it’* Tells N to bring form with her to Labour Ward – (NB no mention N can change her mind)
**N**	still hasn’t let go of consent form – picks up & leaves. No parting words – goodbye, thanks
**Impression**	Distinct feeling that she doesn’t feel heard? wonder if she will go through with Cesarean – whole consultation has felt rushed and a bit chaotic – don’t get any sense of her making a choice. N subsequently confirms to researcher that she’s not sure what to do which was why she was determined not to relinquish the consent form – very unhappy – doesn’t really know how risky a vaginal delivery would be. ** researcher asked HCP later re alternative of vaginal delivery who explained that although she could take her chance with a vaginal delivery it was not really a sensible thing to do in her circumstances

Whereas all HCPs provided some attempt at offering choice and engagement there was variation in the extent to which HCPs used language that was empowering and choice-promoting – *‘it’s very important you know your rights’* and *‘really you haven’t got a choice*’. Some HCPs explicitly acknowledged both the difficulty a woman faced in reconciling uncertain factors to come to a decision and the emotional aspects of making a decision and we observed women whose decision was mostly based on their confidence in their HCP (Table 2).

## Discussion

In this study we prospectively explored how consent takes place by direct observation of consultations regarding ante-natal interventions. Our key findings are: 1) by framing the consultation in terms of the clinical problem HCPs miss the opportunity to assess what really matters to a pregnant woman, 2) discussions around clinical risk do not always reflect current legal guidance, 3) the emphasis on clinical risk can lead to the neglect of other factors which are salient to a particular woman’s decision-making, 4) genuine dialogue influences the interaction between 2) and 3).

Promoting autonomous shared decision-making requires HCPs to appreciate what matters to an individual pregnant woman in a wide and particular way. Yet none of the consultations explicitly addressed a woman’s values and preferences which seems an essential foundation if clinical consultations are to begin to address the legal dictum that HCPs must make comprehensive efforts to ascertain patients’ perspectives on what they want. The whole ethos of patient-centred consultations conceives the patient as an experiencing individual rather than the object of some disease or physiological entity [16] but eliciting patients’ preferences and values effectively is methodologically challenging [17]. Clearly more work is needed on the optimal way of drawing out a pregnant woman’s preferences and values. Our findings suggest that rethinking the framing of consent consultations is key to optimising how autonomous shared decision-making is enacted. At the very least consultations may be improved by the addition of opening questions along the lines of for example, ‘what matters to you most about deciding how to deliver your baby’.

Unsurprisingly, most consultations were dominated by discussion of clinical risks, but women’s engagement varied. Notably, women with previous traumatic experience of giving birth wanted to ensure that their HCP really understood what had happened to them and, importantly, how it had affected them. This need to ‘feel heard’ was often a pre-requisite from the woman’s point of view to her engaging meaningfully in the current consultation so we were interested to observe striking differences between consultations. Whereas some HCPs acknowledged and empathised with the woman and made it clear that they wanted to hear her story, in other, often highly time-pressured consultations, we observed HCPs who wanted to ‘get on’ with the consultation leaving women visibly frustrated and disengaged. By effectively downplaying the woman’s ‘voice’ the HCP’s ‘voice of medicine’ [18] effectively strips away the personal meaning from a woman making it more difficult for HCPs to foster an ‘autonomy through partnership’ decision-making process [1] in which an appreciation of what is material to a particular woman is crucial.

Women are influenced by the attitudes of their obstetricians [19] and surveys indicate that patients value trust over autonomy [20] so we were concerned both to observe consultations where the predominant role adopted by the HCP was that of clinical information provider and consultations in which not all serious risks were disclosed. Overall, our findings seem to undermine the genuine sharing of decision-making and so make HCPs less able to satisfy the legal requirements of materiality. Although HCPs may seek to balance full disclosure with appropriate reassurance and avoidance of undue alarm, such a balancing act points to a conception of autonomy as one of self-determination bounded within the scope of information decided by the HCP. Allied to this our findings suggest that the dialogue and quality of relationship between a woman and her HCP influences a woman’s attitude towards information provided. This is essentially a matter of trust. However it is incumbent on HCPs not to use the engendered trust to subtly ‘encourage’ a particular decision. We were somewhat disconcerted to observe some HCPs selectively finessing the information offered to avoid being ‘*totally alarmist’*. However well-intentioned such clinical approaches are they appear to be at odds with facilitating women’s autonomy.

Many legal cases have endorsed the duty to advise of ‘reasonable’ alternative care options [6, 21, 22]. Our observations of consultations where alternatives were not discussed implies that HCPs are making this decision themselves, presumably on ‘clinical’ grounds. We were concerned to observe several consultations in which the ‘do nothing’ option – usually involving the alternative of a vaginal birth – was not articulated. However justifiable this may be ‘clinically’ it is clearly counter to the spirit of autonomous decision-making and may leave HCPs vulnerable to legal challenge. Other authors [23] have suggested that ‘normal vaginal delivery’ should itself be the subject of a formal consent process so we would caution HCP’s against undiscussed ‘no-brainer’ decision-making.

Following the decision in Montgomery this study involved direct observation of ante-natal consultations. Key strengths of this study were the researchers’ immersion in the clinical environment, the wide clinical range of consultations observed and the ability to clarify observations with participants following the consultation. However, all of the participants in this study had a good command of English and were attending the same ante-natal care facility so it is unknown whether our findings would have differed in non-English speakers and across different clinical sites. Consent is a process and we observed single consultations so we may have overlooked dimensions captured in previous consultations. As with all observational studies, there is the possibility that the presence of a researcher influenced behaviours so although the study revealed many key issues we may have failed to capture some nuances of particular consultations. Whether the results overlap with readers own experiences or indeed have relevance to other antenatal services and patients for example non-pregnant women and men remains open [11] though responses to this paper may strengthen such generalisability.

## Conclusions

We found that post-Montgomery antenatal consent remains pretty much dominated by clinical risk and numerics, and not by what patients and their families consider important, or the value they attach to otherwise sterile numerical probabilities. We have also found that what matters most to women often involves practical, social, and family considerations rather than pure clinical issues. Future initiatives should take this understanding further, to processes and tools that support both patients and their families to discuss their priorities and concerns, and in turn healthcare professionals to be able to provide ‘supported decision making’ and not just longer lists of risks in fear of litigation.

## Data Availability

The fully anonymised datasets used and/or analysed during the current study are available from the corresponding author on reasonable request from authenticated researchers. The study was conducted by HEFCE funded researchers.

## Abbreviation

HCP: Healthcare professional
